# Potential diagnostic markers and biological mechanism for osteoarthritis with obesity based on bioinformatics analysis

**DOI:** 10.1371/journal.pone.0296033

**Published:** 2023-12-21

**Authors:** Qiu Li, Xijie Tang, Weihua Li

**Affiliations:** 1 Department of Cardiovascular, Liyuan Hospital, Tongji Medical College, Huazhong University of Science and Technology, Wuhan, 430077, China; 2 Department of Orthopedics, Wuhan Third Hospital, School of Medicine, Wuhan University of Science and Technology, Wuhan, 430061, China; Concordia University, CANADA

## Abstract

Numerous observational studies have shown that obesity (OB) is a significant risk factor in the occurrence and progression of osteoarthritis (OA), but the underlying molecular mechanism between them remains unclear. The study aimed to identify the key genes and pathogeneses for OA with OB. We obtained two OA and two OB datasets from the gene expression omnibus (GEO) database. First, the identification of differentially expressed genes (DEGs), weighted gene co-expression network analysis (WGCNA), and machine learning algorithms were used to identify key genes for diagnosing OA with OB, and then the nomogram and receiver operating characteristic (ROC) curve were conducted to assess the diagnostic value of key genes. Second, Gene Ontology (GO) and Kyoto Encyclopedia of Genes and Genomes (KEGG) analyses were performed to explore the pathogenesis of OA with OB. Third, CIBERSORT was created to investigate immunocyte dysregulation in OA and OB. In this study, two genes (SOD2, ZNF24) were finally identified as key genes for OA with OB. These two key genes had high diagnostic values via nomogram and ROC curve calculation. Additionally, functional analysis emphasized that oxidative stress and inflammation response were shared pathogenesis of OB and AD. Finally, in OA and OB, immune infiltration analysis showed that SOD2 closely correlated to M2 macrophages, regulatory T cells, and CD8 T cells, and ZNF24 correlated to regulatory T cells. Overall, our findings might be new biomarkers or potential therapeutic targets for OA and OB comorbidity.

## Introduction

Osteoarthritis (OA) is a common, chronic, degenerative joint disease that causes joint stiffness, joint pain, and even disability [1]. It can be a silent illness for a long time before the emergence of representative symptoms and radiography variations, but in such a long-term subclinical phase, the articular damage such as synovial lesions, subchondral osteosclerosis, and cartilage loss could become nonreversible [1–3]. Epidemiological investigations have documented that obesity (OB) is a significant risk factor in the occurrence and progression of OA [4,5]. Among these, a meta-analysis has shown that the risk of knee OA was five times higher in OB patients than in non-obese individuals, and a case-control study has shown that OB patients with OA have higher rates of joint replacement treatments required later in life [6,7]. At present, the incidence of OB continues to increase, thus identifying the shared pathogenesis and therapeutic targets of OB and OA is essential for the prevention and treatment of OA with OB [8].

It was initially believed that the increased susceptibility of OB patients to OA was attributed to the abnormal mechanical load on the joints [9]. However, studies have shown that obese patients also have a higher incidence of OA in non-weight-bearing joints like hands and wrists [10,11]. These findings suggest that OB may cause OA through other pathological mechanisms in addition to purely mechanical factors [12]. Scientists have paid specific attention to the effects of various pro-inflammatory cytokines and adipokines in OA with OB [13–15]. However, it remains unclear whether there are other potential pathologic mechanisms besides immune inflammation. Moreover, the diagnostic value of many genes has not been investigated in OA with OB.

The advancement of sequencing technology and bioinformatics has opened up new opportunities to identify potential biomarkers and their role in different diseases. Additionally, machine learning can be used to excavate pathogenesis at the genetic level and therapeutic targets for a variety of diseases [16]. In our study, we conducted an integrated bioinformatics analysis and machine learning to identify the shared key genes and molecular mechanisms for OA and OB, which may provide clues for exploring the genetic etiology and combined therapeutic strategy for this comorbidity.

## Materials and methods

### Microarray data

**Fig 1:**
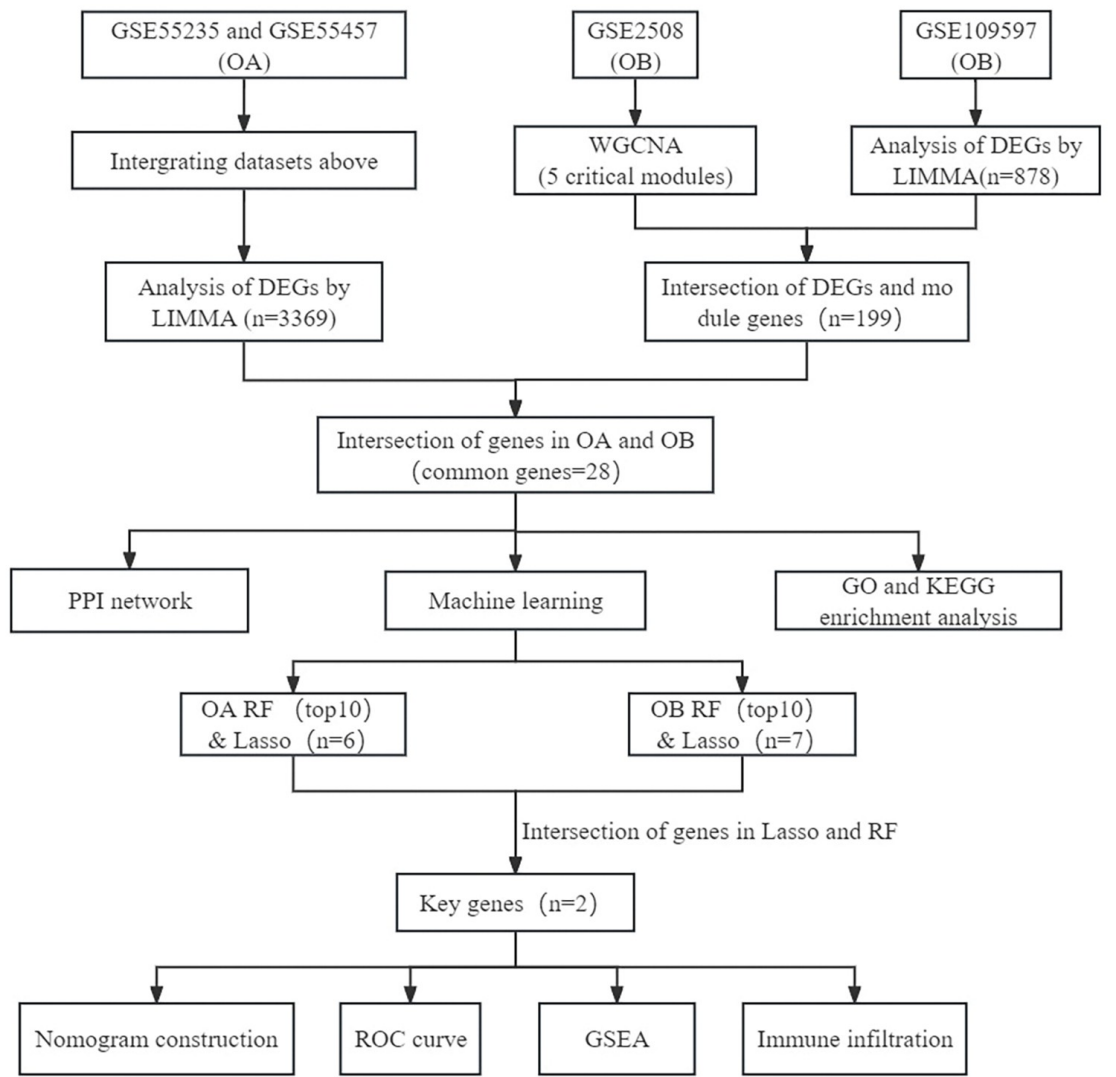
Study flowchart. OA, osteoarthritis; OB, obesity; WGCNA, weighted gene co-expression network analysis; DEGs, differentially expressed genes; PPI, protein–protein interaction; GO, Gene ontology; KEGG, Kyoto Encyclopedia of Genes and Genomes; RF, random forest; LASSO, least absolute shrinkage and selection operator; ROC, receiver operating characteristic; GSEA, Gene set enrichment analysis.

The study flowchart is depicted in Fig 1. OA-related datasets (GSE55235 and GSE55457) were downloaded from the Gene Expression Omnibus (GEO) database (http://www.ncbi.nlm.nih.gov/geo), and the samples of OA and health were all taken from human synovial membrane. Two OB-related datasets (GSE2508, GSE109597) were also selected from the GEO database. Among them, the samples of GSE2508 were derived from human subcutaneous adipose tissue and the samples of GSE109597 were derived from human peripheral blood. All dataset information is outlined in detail in Table 1.

**Table 1:** Basic information of gene expression omnibus (GEO) datasets used in the study.

Disease	GSE series	Platform	Sample size	Source types
OA	GSE55235	GPL96	10 OA and 10 Normal	Synovial membrane
OA	GSE55457	GPL96	10 OA and 10 Normal	Synovial membrane
OB	GSE2508	GPL95	20 OB and 19 Normal	Subcutaneous adipose
OB	GSE109597	GPL570	41 OB and 43 Normal	whole blood

OA, osteoarthritis; OB, obesity.

### Data processing and differentially expressed genes identification

The two OA datasets (GSE55235 and GSE55457) were merged into a metadata cohort using “affy” in R, and the Bioconductor “SVA” R package was applied to eliminate the batch effect [18]. Differentially expressed genes (DEGs) of the OA merged dataset and OB dataset (GSE109597) were identified with the selection criteria of P <0.05 and |log2FC| ≥1 via the “limma” package in R. The results of DEGs were visualized by the volcano plot and heatmap.

### Weighted gene co-expression network analysis and module gene selection

Using the “WGCNA” package, we performed weighted gene co-expression network analysis (WGCNA) analysis to identify critical modules for OB (GSE2508). WGCNA is a system biology strategy that can construct co-expressed gene modules and explore the correlation between genes and diseases [19]. First, the median absolute deviation (MAD) of each gene was determined, and 50% of genes with the smallest MAD were removed. Second, the missing and outlier samples were excluded using the Hclust function and goodSamplesGenes function. Third, the adjacency was computed by co-expression similarity-derived “soft” thresholding power (β) and then converted into a topological overlap matrix (TOM) and the corresponding dissimilarity (1−TOM). Fourth, average linkage hierarchical clustering was used to conduct a clustering dendrogram of the TOM matrix, and similar gene expressions were divided into different modules with a minimum gene group size (n = 100). Fifth, the correlation between the phenotype and each module was assessed, where the correlation modules with p<0.05 were defined as critical modules. Finally, the eigengene network was visualized.

### Functional enrichment analysis

To recognize the shared biological process and signaling pathway, we conducted Gene Ontology (GO) and Kyoto Encyclopedia of Genes and Genomes (KEGG) enrichment analyses for the common genes of OA and OB using the “clusterProfiler” package in R. Additionally, Gene Set Enrichment Analysis (GSEA) was chosen to discover the underlying molecular mechanisms of key genes by “clusterProfiler” package. The results of enrichment analysis with a P-value <0.05 was considered significant in statistics and were visualized via the Sangerbox platform (http://vip.sangerbox.com/).

### Protein–protein interaction network construction

To excavate interactions among protein-coding genes, we utilized the STRING database (http://string-db.org/) to construct a protein-protein interaction (PPI) network for common genes of OA and OB. PPI pairs with an interaction score greater than 0.15 were extracted.

### Machine learning

Two machine learning algorithms, random forest (RF) and least absolute shrinkage and selection operator (LASSO) algorithms, were adopted to further identify key genes for diagnosing OA with OB using “randomForest” and “glmnet” R packages [17]. RF is a sensitive and specific approach for predicting continuous variables and providing forecasts without apparent variations, which has the advantage of no limits on variable conditions [18]. LASSO is a regression method for selecting a variable to improve the predictive accuracy and comprehensibility of a statistical model [19]. The intersection genes of LASSO and RF were considered key genes for OA with OB diagnosis.

### Nomogram construction and receiver operating characteristic evaluation

Based on key genes, the nomograms and receiver operating characteristic (ROC) curve are valuable for predicting the clinical diagnosis of OA with OB. In the nomograms, the “points” represent the scores assigned to key genes, and the “total points” are the sum of all the scores [20]. In the ROC, the area under the curve (AUC) and 95% CI were calculated to quantify the diagnostic value of key genes for disease, and an AUC > 0.7 was considered the ideal diagnostic value [21].

### Immune infiltration analysis

CIBERSORT is a deconvolution algorithm for recognizing the proportion of diverse immunocytes in the microenvironment according to tissue gene expression profiles [22,23]. Immune cell infiltration analysis was performed using the “Cibersort” R package, and the proportion of 22 immune cells in different samples was visualized by barplot and box diagram. Additionally, the associations between 22 immune cells and key genes were assessed and were visualized by a lollipop chart.

## Results

### DEGs identification in OA and OB

**Fig 2:**
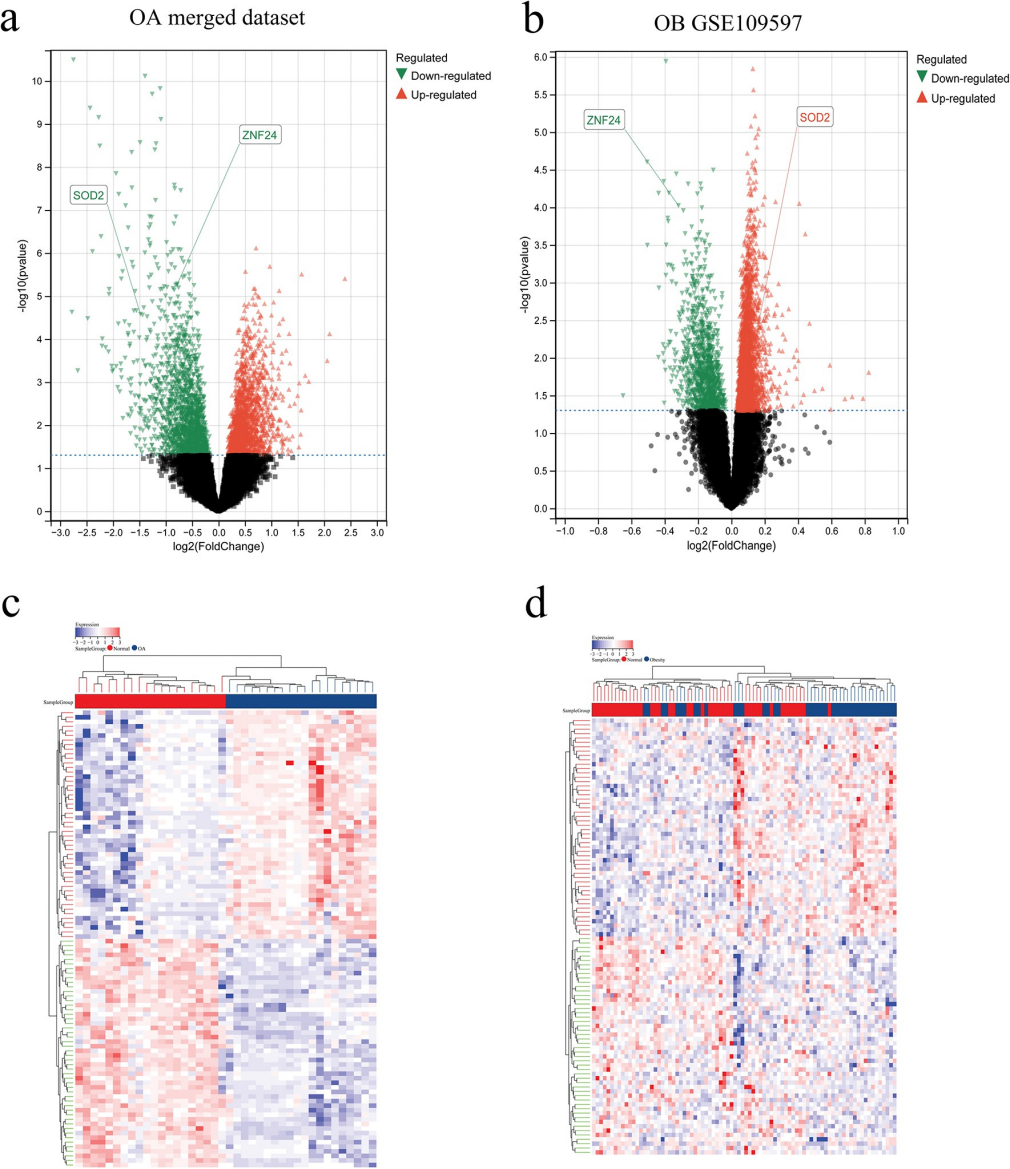
DEGs identified from the OA merged dataset and OB dataset (GSE109597). (a) Volcano plot of DEGs in OA merged dataset, red: upregulated genes; green: downregulated genes. (b) Volcano plot of DEGs in OB dataset (GSE109597), red: upregulated genes; green: downregulated genes. (c) Heatmap of DEGs in OA merged dataset. (d) Heatmap of DEGs in OB dataset (GSE109597). OA, osteoarthritis; OB, obesity.

Using the limma R package, a total of 3369 DEGs were identified from the OA merged dataset, with 1577 genes upregulated and 1792 genes downregulated (Fig 2A and 2B). The OB dataset (GSE109597) had 3880 DEGs, with 2731 genes upregulated and 1149 genes downregulated (Fig 2C and 2D).

### Identification of critical modules in OB

**Fig 3:**
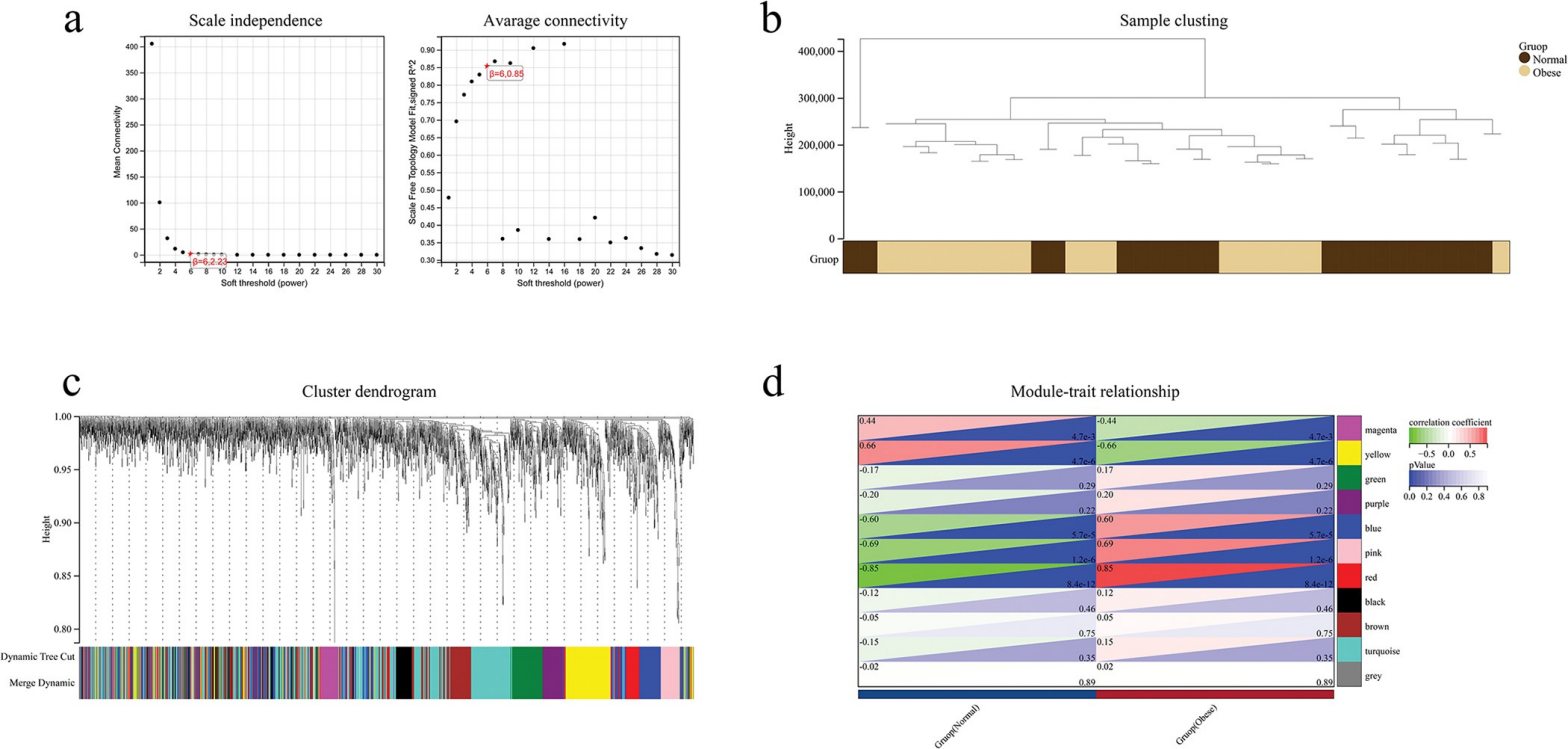
Module genes identified via WGCNA in OB dataset (GSE2508). (a) β = 6 is selected as the soft threshold with the combined analysis of scale independence and average connectivity. (b) Clustering dendrogram of the OB and normal samples. (c) Gene co-expression modules represented by different colors under the gene tree. (d) Heatmap of the association between modules and OB.

Using WGCNA analysis, a total of 11 modules were detected in GSE2508 based on β = 6 (scale-free R^2^ = 0.85) as the“soft” threshold, and each module was labeled with a unique color (Fig 3A and 3B). Fig 3C depicts the clustering dendrogram of OB and control samples. Fig 3D showed that 5 modules were significantly associated with OB and were selected as critical modules. Among these 5 critical modules, 3 modules (blue: r = 0.60, p = 5.7e−5; pink: r = 0.69, p = 1.2e−6; red: r = 0.85, p = 8.4e−12) were positively related to OB, while the other 2 modules (magenta: r = -0.44, p = 4.7e−3; yellow: r = -0.66, p = 4.7e−6) were negatively related to OB (Fig 3D). Then all critical modules were left for further analysis.

### Enrichment analysis of the common genes in OB and OA

**Fig 4:**
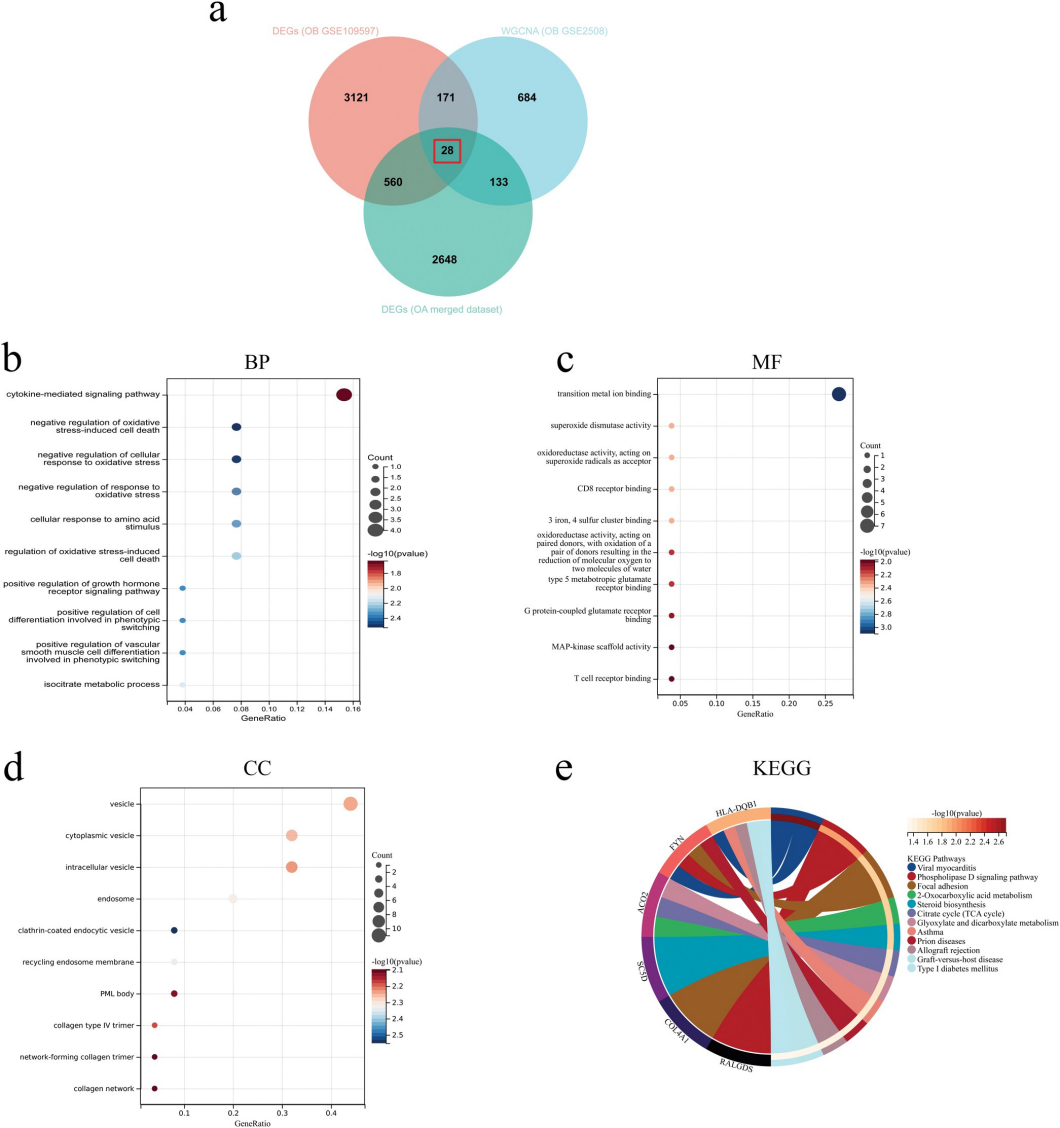
Enrichment analysis of common genes of OA and OB. (A) Venn diagram of the intersection genes of OA and OB. (b-d) GO analysis of 28 common genes, including BP, CC, and MF, respectively. (e) KEGG analysis of 28 common genes. OA, osteoarthritis; OB, obesity; DEGs, differentially expressed genes; WGCNA, weighted gene co-expression network analysis; GO, Gene ontology; BP, biological process; CC, cellular component; MF, molecular function; KEGG, Kyoto Encyclopedia of Genes and Genomes.

28 common genes of OA and OB that were extracted from the intersection of the DEGs for the OA merged dataset, the critical modules genes for the OB dataset GSE2508, and the DEGs for the OB dataset GSE109597 (Fig 4A). The KEGG enrichment analysis revealed that 28 common genes were primarily enriched in the “Phospholipase D signaling pathway,” “2-Oxocarboxylic acid metabolism,” and “Glyoxylate and dicarboxylate metabolism” (Fig 4E and S4 Table). Furthermore, the results of GO analysis showed that the common genes were enriched in “cytokine-mediated signaling pathway,” “regulation of oxidative stress,” and “cellular response” (BP); “transition metal ion blinding, “superoxide dismutase activity,” and “CD8 receptor binding” (MF); and “vesicle,” “cytoplasmic vesicle,” “intracellular vesicle,” and “endosome” (CC) (Fig 4B, 4C and 4D and S1–S3 Tables).

### Identification of key genes via machine learning

**Fig 5:**
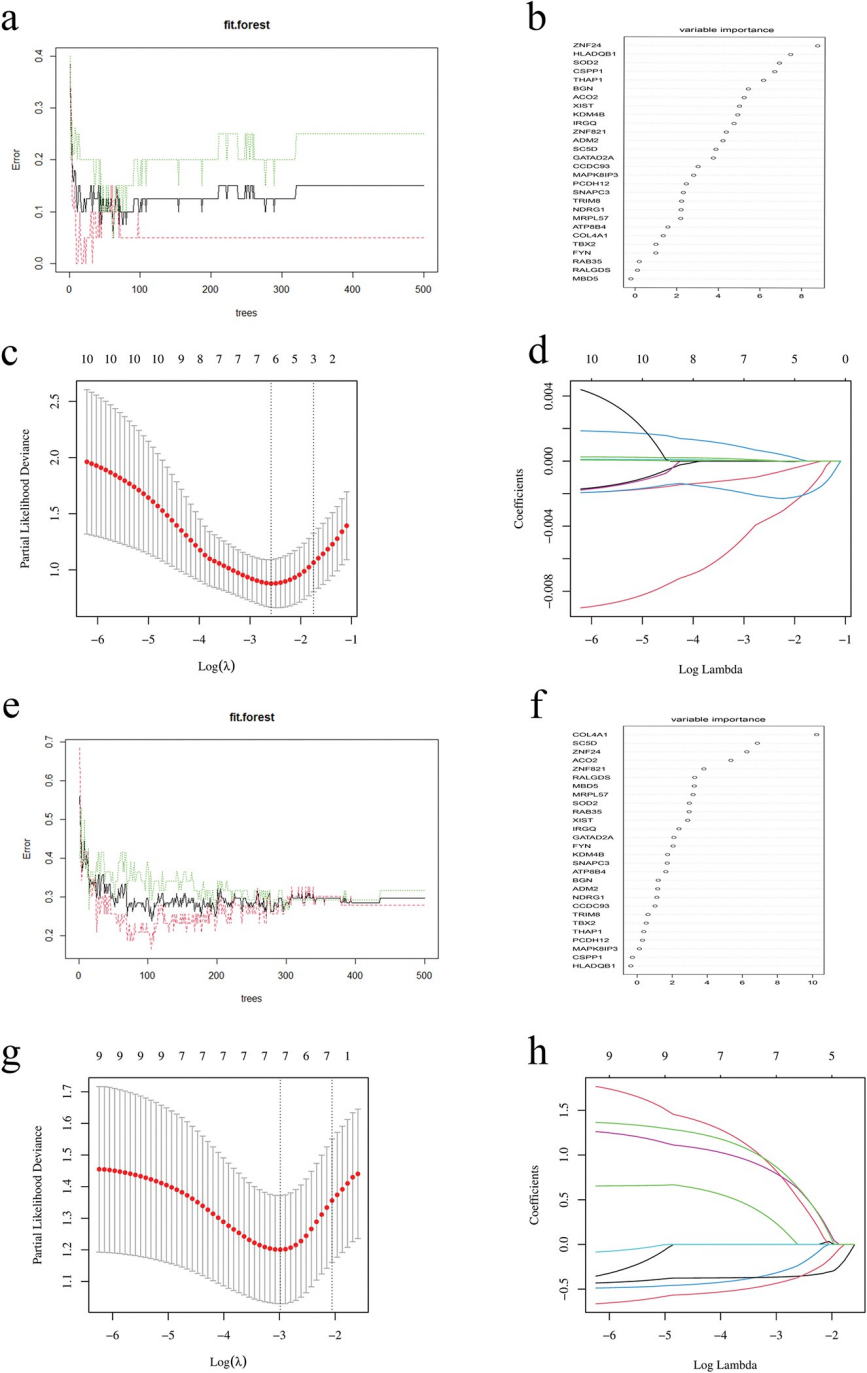
Machine learning in screening key diagnostic biomarkers for OA with OB. (a) The RF shows the error and the importance of 28 common genes in OA. (c, d) Biomarkers screening in the LASSO model, and the number of genes (n = 6) corresponding to the lowest point of the curve is the most suitable for OA diagnosis. (e, f) The RF shows the error and the importance of 28 common genes in OB (GSE109597). (g, h) Biomarkers screening in the Lasso model, and the number of genes (n = 7) corresponding to the lowest point of the curve is the most suitable for OB diagnosis. RF, random forest; LASSO, least absolute shrinkage and selection operator; OA, osteoarthritis; OB, obesity.

Key genes were further identified from common genes by RF algorithms and LASSO regression in both OA and OB datasets. In the OA merged dataset, the RF algorithm ranked the genes based on the importance of each gene, and the LASSO regression identified six potential biomarkers (KDM4B, XIST, ACO2, SOD2, HLA-DQB1, and ZNF24) (Fig 5A and 5B). Additionally, considering that the substances secreted by adipose tissue are primarily dependent on blood metabolism to affect OA, we conducted an RF algorithm and LASSO regression for the OB dataset GSE109597, whose samples were from human peripheral blood. In GSE109597, the RF algorithm ranked the importance of 28 common genes and the LASSO regression identified seven potential biomarkers (SOD2, MRPL57, MBD5, ZNF821, ZNF24, SC5D, SC5D, and COL4A1) (Fig 5C and 5D). The intersection of the top 10 most important genes from the RF and the potential biomarkers from LASSO was visualized via the Venn diagram (S1 Fig), and two genes (SOD2 and ZNF24) were identified as key genes. Moreover, based on the PPI network of 28 common genes, we found that SOD2 and ZNF24 could interact with each other through CSPP1, and the 28 genes were ranked by node numbers (S1 Fig).

### Diagnostic value assessment of key genes

**Fig 6:**
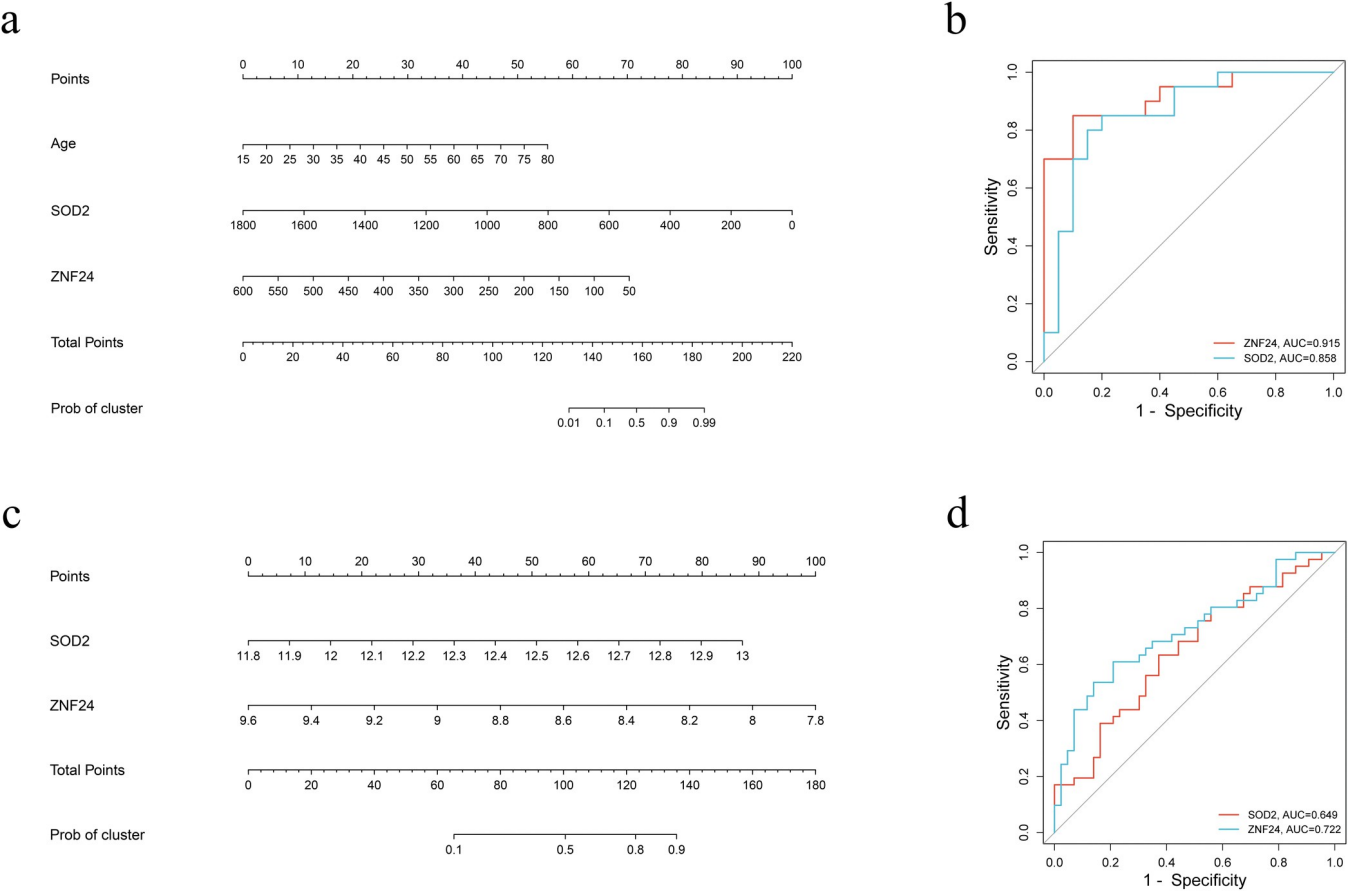
Nomogram construction and the diagnostic value evaluation. (a) The visible nomogram for diagnosing OA. (b) The ROC curves of SOD2 and ZNF24 in OA. (c) The visible nomogram for diagnosing OB (GSE109597). (d) The ROC curves of SOD2 and ZNF24 in OB (GSE109597).

The nomogram was constructed based on the two key genes, and the ROC curve was established to assess the diagnostic specificity and sensitivity of the nomogram. The results of the nomogram suggested that SOD2 and ZNF24 had significant effects on both OA and OB (Fig 6A and 6C). The results of ROC curve also indicated that SOD2 (OA: AUC 0.85, 95%CI 0.73–0.98; OB GSE109597: AUC 0.64, 95%CI 0.52–0.76) and ZNF24 (OA: AUC 0.71, 95%CI 0.53–0.88; OB GSE109597: AUC 0.72, 95% CI 0.61–0.83) had a high diagnostic value for OA and OB (Fig 6B and 6D).

### GSEA analysis of key genes

**Fig 7:**
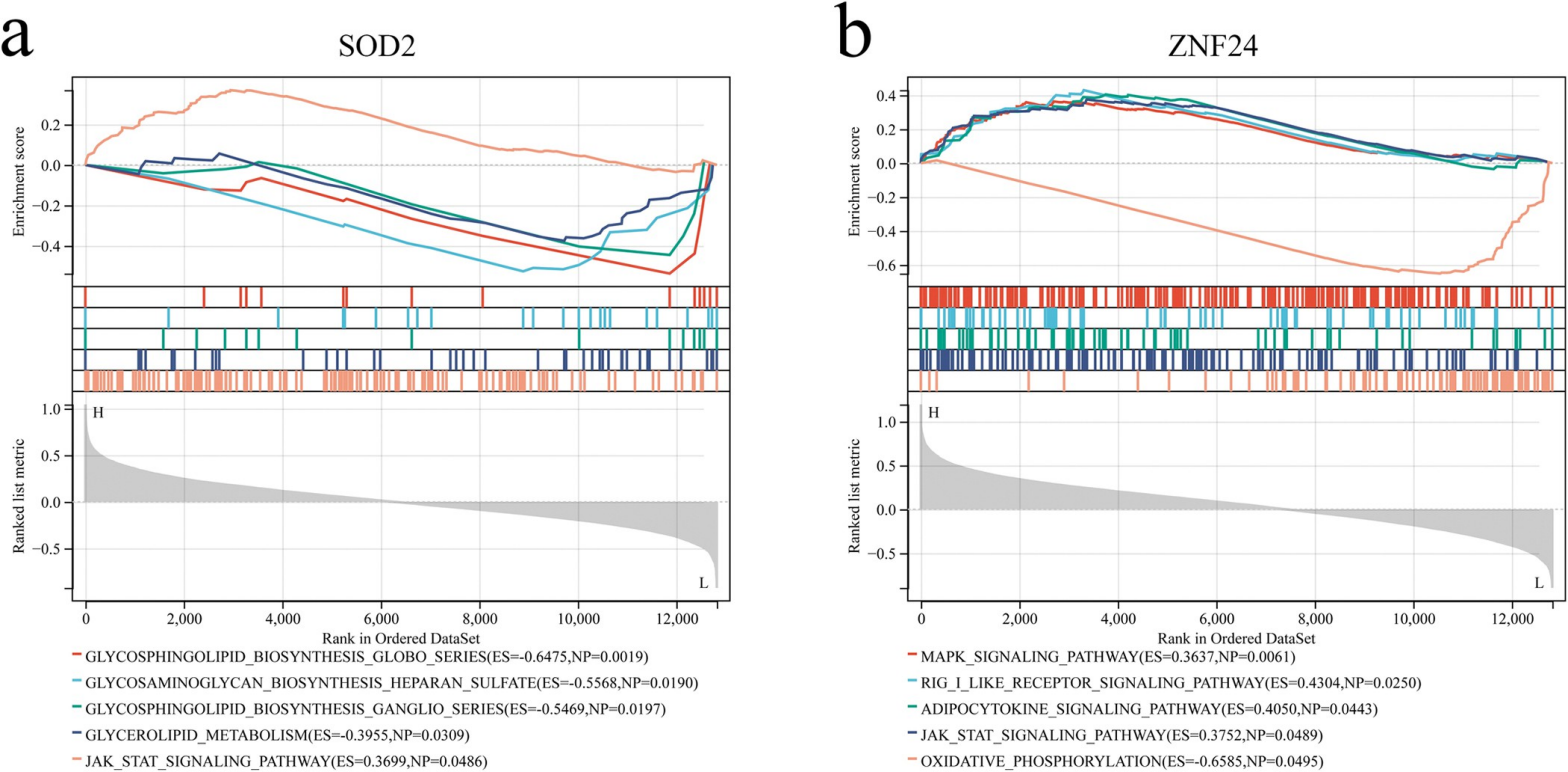
The functional enrichment analysis of SOD2 and ZNF24 in OA. (a) The main enrichment pathway of SOD2. (b) The main enrichment pathway of ZNF24.

GSEA analysis was conducted to discover the underlying molecular mechanisms of key genes in OA. GSEA suggested that SOD2 was mainly involved in biosynthesis, metabolism, and JAK-STAT signaling pathways (Fig 7A). Moreover, QRFPR was mainly enriched in MAPK, RIG-I-like receptor, adipocytokine, JAK-STAT, and oxidative phosphorylation signaling pathways (Fig 7B).

### Correlation between key genes and immunocyte infiltration levels

**Fig 8:**
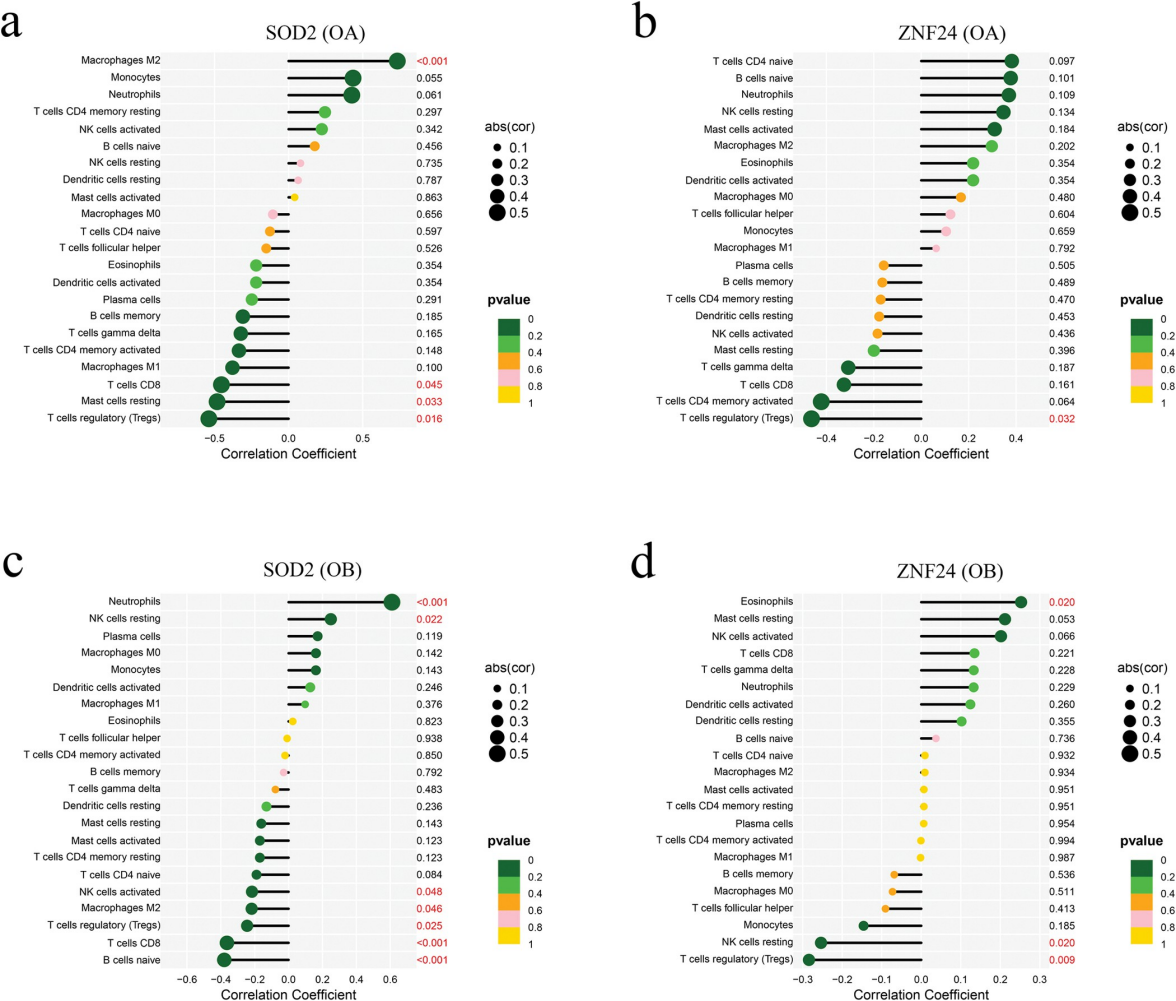
Correlation between key genes and immunocyte in OA and OB. (a) Correlation between SOD2 and immunocyte in OA and normal samples. (b) Correlation between ZNF24 and immunocyte in OA and normal samples. (c) Correlation between SOD2 and immunocyte in OB and normal samples. (d) Correlation betweenZNF24 and immunocyte in OA and normal samples.

Since the immuno-inflammatory response is considered to be the most important modulator of OA with OB, the CIBERSORT approach was performed to better elucidate the immune regulation of OA and OB. The proportion of 22 kinds of immune cells in each sample of OA and OB is displayed in the barplot and box plot (S2 Fig). Regarding the OA and control groups, SOD2 was correlated with M2 macrophages, CD8 T cells, mast cells resting, and regulatory T cells, and ZNF24 was correlated with regulatory T cells (Fig 8A and 8B). Additionally, between normal samples and OB samples, SOD2 was correlated with neutrophils, NK cells resting, NK cells activated, M2 macrophages, regulatory T cells, CD8 T cells, and naive B cells, and ZNF24 was correlated with eosinophils, NK cells resting, and regulatory T cells (Fig 8C and 8D). Our findings suggested that SOD2 may be involved in the progression of OA with OB via regulating M2 macrophages, regulatory T cells, and CD8 T cells, while ZNF24 only via regulating regulatory T cells.

## Discussion

OA is one of the major diseases that cause activity restriction in adults, and its main risk factors are well known including genetics, age, female sex, sedentary, major joint injury, and so on [24,25]. Of these risk factors, OB is considered a prominent one. Previous studies have shown that OB and OA share some pathogenic pathways in addition to purely biomechanical effects [13]. However, these studies tended to take a clinical approach were unable to reveal the molecular mechanism at the genetic level, and did not propose biomarkers and targeted therapy for comorbidity patients.

In this study, we integrated bioinformatics analysis and machine learning methods to identify key genes and to evaluate their diagnostic value for OA patients with OB. The most noteworthy discovery is that two genes (SOD2 and ZNF24) were identified as key genes with high diagnostic value, and they could interact with each other through intermediate molecules. Among them, immune infiltration analysis suggested that SOD2 mediated the progression of OA in OB patients by regulating M2 macrophages, regulatory T cells and CD8 T cells, and ZNF24 by regulating regulatory T cells. Additionally, we conducted a series of enrichment analyses, and the results revealed that oxidative stress also plays a prominent role in the pathogenesis of OA in OB patients in addition to immune responses.

Oxidative stress is a cellular response caused by an imbalance between reactive oxygen species (ROS) production and antioxidant defenses, which can lead to cellular damage and in some cases cell death [26,27]. Evidence is mounting that human OB is a state of chronic oxidative stress of the whole body since it perturbs the antioxidant defense system and reduces the activity of protective antioxidant enzymes in tissues [28–30]. Moreover, the chondrocytes and synovial fibroblasts of OA can also produce large amounts of ROS in response to biomechanical or biochemical stimuli, thus aggravating oxidative stress and cell damage in OA [31–33]. Therefore, Oxidative stress maybe not only the common pathological mechanism of OA and OB but also an important player for OA development affected by OB. Notably, ROS and inflammation are interdependent and inseparable, and the contribution of inflammatory responses to OA development in OB patients is beyond doubt [34,35]. Research showed that proinflammatory cytokines (TNFα, IL-1β, and IL-6) can induce ROS accumulation in joint cells, while ROS could participate in the processes of the inflammatory response by triggering specific intracellular pathways such as elicited by mitogen-activated protein kinases (MAPK), nuclear Factor Kappa B (NFκB), JAK/STAT and Wnt pathway [36].

Superoxide dismutase 2 (SOD2), an effective scavenging enzyme of ROS, can efficiently convert superoxide to the less reactive hydrogen peroxide (H2O2) that can freely diffuse across the mitochondrial membrane [37]. A decrease in SOD2 expression can lead to increased inflammation in metabolically active adipose tissue, resulting in damage to surrounding tissue [38–40]. Jenny L Scott et al found that SOD2 was abundantly expressed in human healthy cartilage but was markedly down-regulated in end-stage OA cartilage, and the depletion of SOD2 gave an increased ROS but a decreased collagenase expression in OA [41]. Subsequent findings indicate that SOD2 depletion could lead to mitochondrial dysfunction, and further contribute to oxidative damage of joint cells in OA [42]. Therefore, developing new therapeutic approaches to enhance SOD2 activity may be beneficial in slowing the progression of the comorbidities of OA and OB.

Zinc finger protein 24 (ZNF24; also known as KOX17, Zfp191, or ZNF191) is the novel biomarker identified in our study in diagnosing OA in OB patients. It belongs to a member of the Kruppel-like zinc finger transcription factor family, possessing four C2H2 zinc finger domains at the C-terminus for DNA binding and one SCAN domain at the N-terminus [43–45]. ZNF24 is a key regulator of normal development of tissues, which is involved in the regulation of the proliferation, migration, differentiation, and invasion of cells of different lineages [46–49]. In addition to regulating normal cells, ZNF24 has been shown to have significant effects on the initiation and progression of cancer and angiogenesis [50–53]. There have been no studies on the effects of ZNF24 on OA or OB, while research indicated that ZNF24 negatively regulate the signaling activity of the NF-κB pathway and Wnt pathway, and these two pathway has been reported to be involved in physiological processes of inflammatory responses [50,54]. Additionally, our research also observed that ZNF24 was mainly enriched in oxidative stress and inflammatory signaling pathways, the function of ZNF24 in OA and OB is worthy of further study.

There is one major strength in our work, which was that the samples regarding the OB dataset used as the main analysis are from peripheral blood; therefore, we only need to collect peripheral blood from OB patients and evaluate the expression of the two genes to infer the probability of OA incidence in OB patients via nomogram. There are also some limitations to be acknowledged in our study. First, our study was only able to explore the shared diagnostic genes of OA and OB, but it can not explain whether the influence of the key genes on OA was mediated by OB due to the lack of dataset on OA and OB comorbidities. Second, the influence and exact mechanisms for the two key genes mediating OA and OB comorbidity need further investigation. Lastly, we lack in vivo or in vitro experiments to validate our results.

## Conclusion

Our study systematically discovered two key genes (SOD2 and ZNF24) and provided the nomogram for diagnosing OA with OB by various bioinformatics analyses and machine learning algorithms. We also illustrated the associations of immunocytes with key genes in OA and OB. Moreover, we found that oxidative stress is a potential mechanism for worsening OA in OB patients. These findings may provide new biomarkers or potential therapeutic targets for OA and OB comorbidity.

## Supporting information

S1 Table The GO biological process analyses of 28 common genes of OA and OB. (XLSX)

S2 Table The GO cellular component analyses of 28 common genes of OA and OB. (XLSX)

S3 Table The GO molecular function analyses of 28 common genes of OA and OB. (XLSX)

S4 Table The KEGG analyses of 28 common genes of OA and OB. (XLSX)

S1 Fig PPI network of 28 common genes in OA and OB. (TIF)

S2 Fig Immune cell infiltration analysis between OA and OB. (TIF)
