# Supplementary figures and images for: Potential diagnostic markers and biological mechanism for osteoarthritis with obesity based on bioinformatics analysis
Source: PLoS One. 2023 Dec 21;18(12):e0296033. doi: 10.1371/journal.pone.0296033 (PMC10735003; doi:10.1371/journal.pone.0296033)

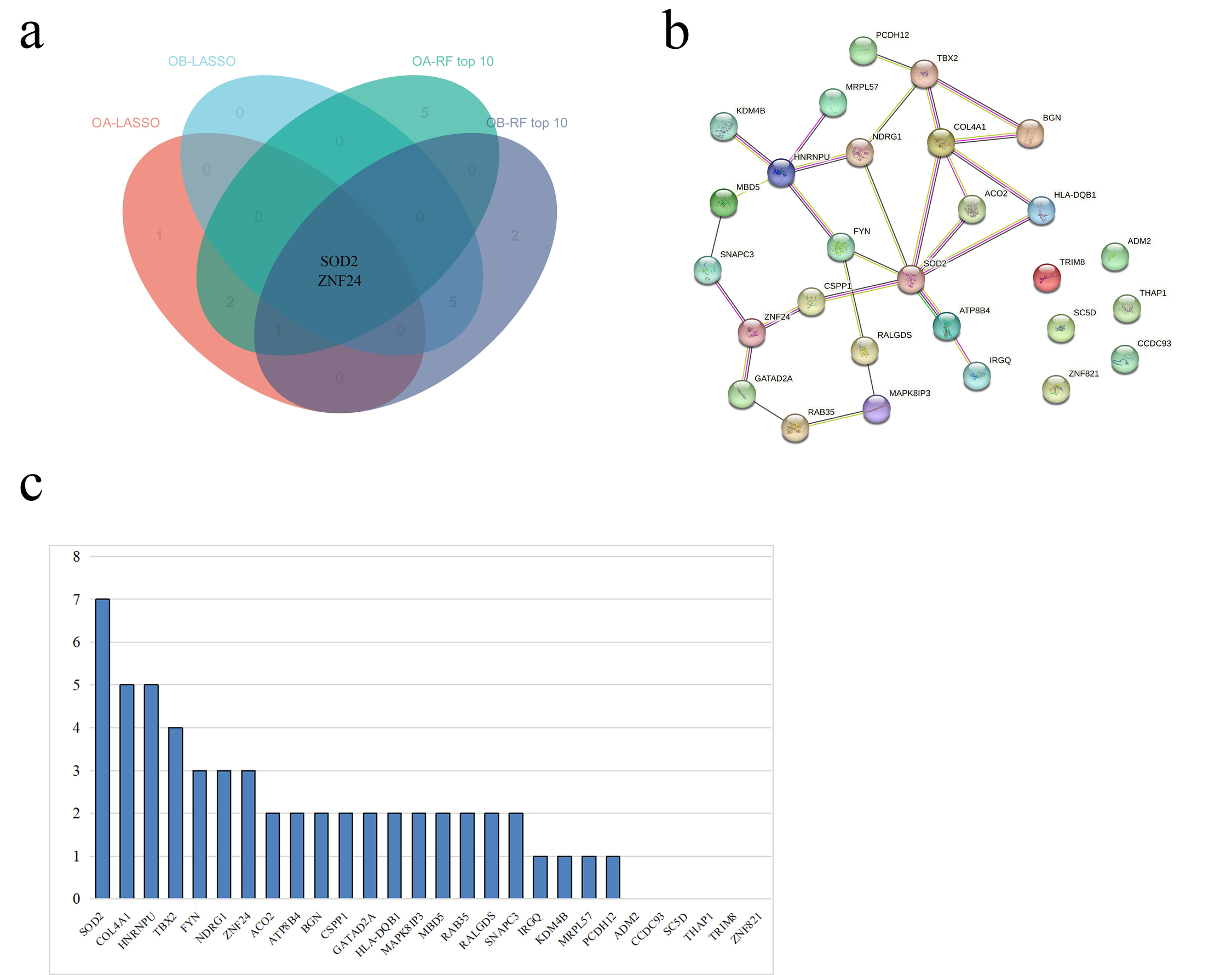

Supplement: S1 Fig — (TIF) [file pone.0296033.s005.tif]

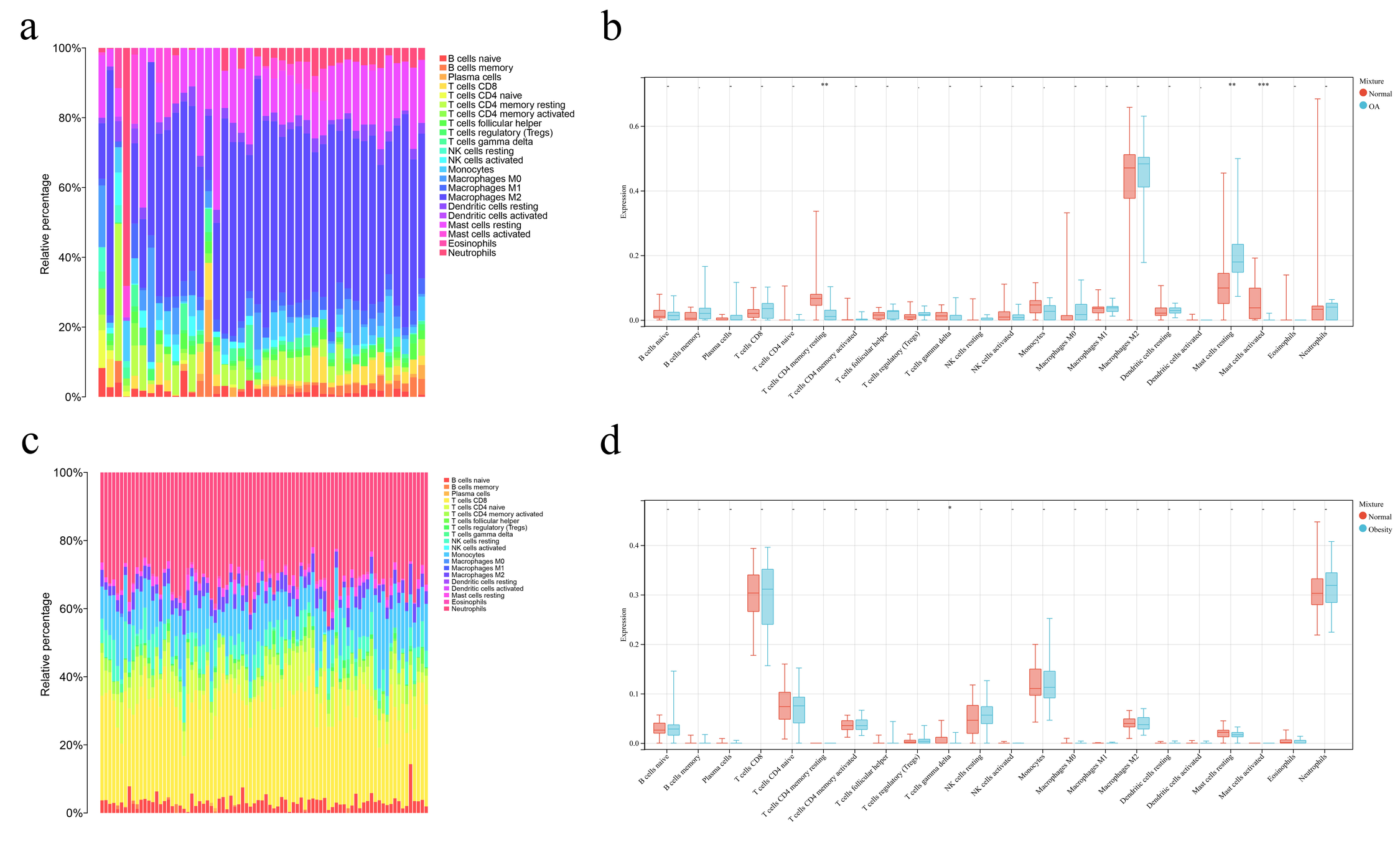

Supplement: S2 Fig — (TIF) [file pone.0296033.s006.tif]
